# Fatty Degeneration of the Autochthonous Muscles Is Significantly Associated with Incidental Non-Traumatic Vertebral Body Fractures of the Lower Thoracic Spine in Elderly Patients

**DOI:** 10.3390/jcm12144565

**Published:** 2023-07-08

**Authors:** Jan-Christoph Backhauß, Olav Jansen, Hans-Ulrich Kauczor, Sam Sedaghat

**Affiliations:** 1 Department for Radiology and Neuroradiology, University Hospital Schleswig-Holstein Campus Kiel, 24105 Kiel, Germany; 2 Department of Diagnostic and Interventional Radiology, University Hospital Heidelberg, 69120 Heidelberg, Germany

**Keywords:** spine, osteoporosis, fracture, muscle degeneration, computed tomography

## Abstract

Purpose: We investigated loco-regional degenerative changes’ association with incidentally found non-traumatic vertebral body fractures of the lower thoracic and lumbar spine in older patients. **Methods:** The patient collective included patients in the age range of 50 to 90 years. Vertebral bodies from T7 to L5 were included. Vertebral body fractures were classified according to Genant. The following loco-regional osseous and extra-osseous degenerative changes were included: osteochondrosis, spondylarthritis, facet joint asymmetries, spondylolisthesis, scoliosis as well as fatty degeneration and asymmetry of the autochthonous back muscles. Patients with traumatic and tumor-related vertebral body fractures were excluded. Non-traumatic fractures of the lower thoracic and lumbar spine were evaluated separately. The Mann–Whitney U-test was used, and relative risks (RRs) were calculated for statistics. Pearson’s correlations (Rs) were used to correlate grades of degenerative changes and fracture severities. **Results:** 105 patients were included. Fatty deposits in the autochthonous muscles of the lower thoracic and the lumbar spine were associated with non-traumatic vertebral body fractures in the lower thoracic spine (*p* = 0.005, RR = 4.92). In contrast, muscle fatness of the autochthonous muscles was not a risk factor for lumbar spine fractures (*p* = 0.157, RR = 2.04). Additionally, we found a moderate correlation between fatty degeneration of the autochthonous muscles and the severity of fractures in the lower thoracic spine (RR = 0.34, *p* < 0.001). The other degenerative changes did not present any significant difference or correlation between the evaluated groups. **Conclusions:** Fatty degeneration of the autochthonous spinal musculature is associated with incidentally found non-traumatic fractures of the lower thoracic spine.

## 1. Introduction

As vertebral compression fractures can occur unnoticed, cause spinal deformity, and may require further surgical or medical treatment, it is of interest to see if there are any correlations with the degenerative spinal changes that radiologists can highlight in their daily reports of common CT scans [1]. This could help avoid the summarization of degenerative changes often seen in radiological reports.

Degenerative changes in the spine include a variety of abnormalities, both osseous and extra-osseous, which are constantly changing [2]. These degenerations, such as osteochondrosis or spondylarthrosis, can cause low back pain or be completely asymptomatic [2,3]. Vertebral wedge compression fractures, both degenerative and osteoporotic, often occur over time without the patient experiencing significant back pain.

Extra-osseous degeneration mainly involves fatty transformation and volume loss of the autochthonous back muscle, which can cause back pain [4]. Strengthening the back muscles with specific exercises can reduce pain, improve movement and even aid recovery after a vertebral fracture [5]. In addition, practice is also used to prevent fall-related vertebral fractures, with various training methods including improving balance and weight bearing, all to achieve more significant support and protection with strong autochthonous back muscles [6]. These clinical aspects highlight the importance of analyzing and assessing the autochthonous back musculature on conventional radiological imaging.

The current leading imaging modality for visualizing and classifying all kinds of degenerative changes in the spine is magnetic resonance imaging (MRI) which is closely followed by computed tomography (CT) [7,8,9]. For the detailed characterization of vertebral fractures, CT is state of the art, whereas MRI is mainly used to determine the age of fractures [10,11]. The use of CT to analyze autochthonous back muscles, including general structure, volume, asymmetry, and fat deposition, is part of many studies, especially as fat infiltration is visible as a decrease in density in Hounsfield units [12,13].

The spine is often included in radiological imaging, although mainly not the primary target of the various imaging examinations performed by radiologists. When comparing imaging techniques, incidental findings are the most common in CT scans of patients [14]. These include mainly vascular, pulmonary, hepatobiliary, renal, gastrointestinal, and, when considering the musculoskeletal system, besides common degenerative findings, non-traumatic fractures of the vertebral bodies [14,15]. Degenerative changes can account for many different lesions and are often summarized or not mentioned at all. By associating these changes with non-traumatic vertebral body fractures, their impact on the spine and further treatment of the patient can be analyzed.

This study investigates the association of several loco-regional degenerative changes in the lower thoracic and lumbar spine to non-traumatic vertebral body fractures incidentally found during non-spine-targeted CT examinations.

## 2. Materials and Methods

### 2.1. Patients and Imaging

Patients aged 50 to 90 years who underwent outpatient, non-spine-targeted CT examination at our institution within a pre-defined time frame were included. To achieve a normal distribution of patients according to age ranges, almost equal proportions of patients aged 50–60, 61–70, 71–80, and 81–90 years were built. Additionally, we achieved an almost equal fracture distribution within the age ranges, minimizing the possibility of bias caused by patients’ age. Only patients with a complete depiction of spine levels T7 to L5 were included due to the prevalence of vertebral fractures in these areas and generally the highest number of incidental findings on abdominal CT scans [14,15,16,17]. Only mobile patients with no movement-restricting pre-conditions were included.

Patients with tumor-related changes in the spine, both primary and metastatic, acute inflammatory changes such as spondylodiscitis or implanted foreign material (e.g., dorsal spondylodesis, intervertebral disc interpositions, vertebra cages, and kyphoplasty) were excluded from this study. In addition, patients with acute trauma and a consecutive vertebral fracture were excluded, intentionally leaving only patients with non-traumatic fractures. Patients with known vertebral body fractures were deliberately excluded, as awareness of the fracture could have altered the patient’s mobility, resulting in a change in back muscle structure. Additionally, immobile patients were also excluded to reduce the possible effect of immobilization on the evaluated degenerative changes.

Finally, 25 patients with at least one non-traumatic fracture of the lower thoracic and lumbar vertebral body were identified, and 80 control patients without non-traumatic fractures in the same levels were included in this Institutional Review Board (IRB)-approved study. The term ‘non-traumatic fracture’ refers to a fracture without acute traumatic events that would bring the patient to the hospital. Accordingly, the patients were unaware of their fractures and did not seek hospital admission or fracture-related therapy.

**Figure 1:**
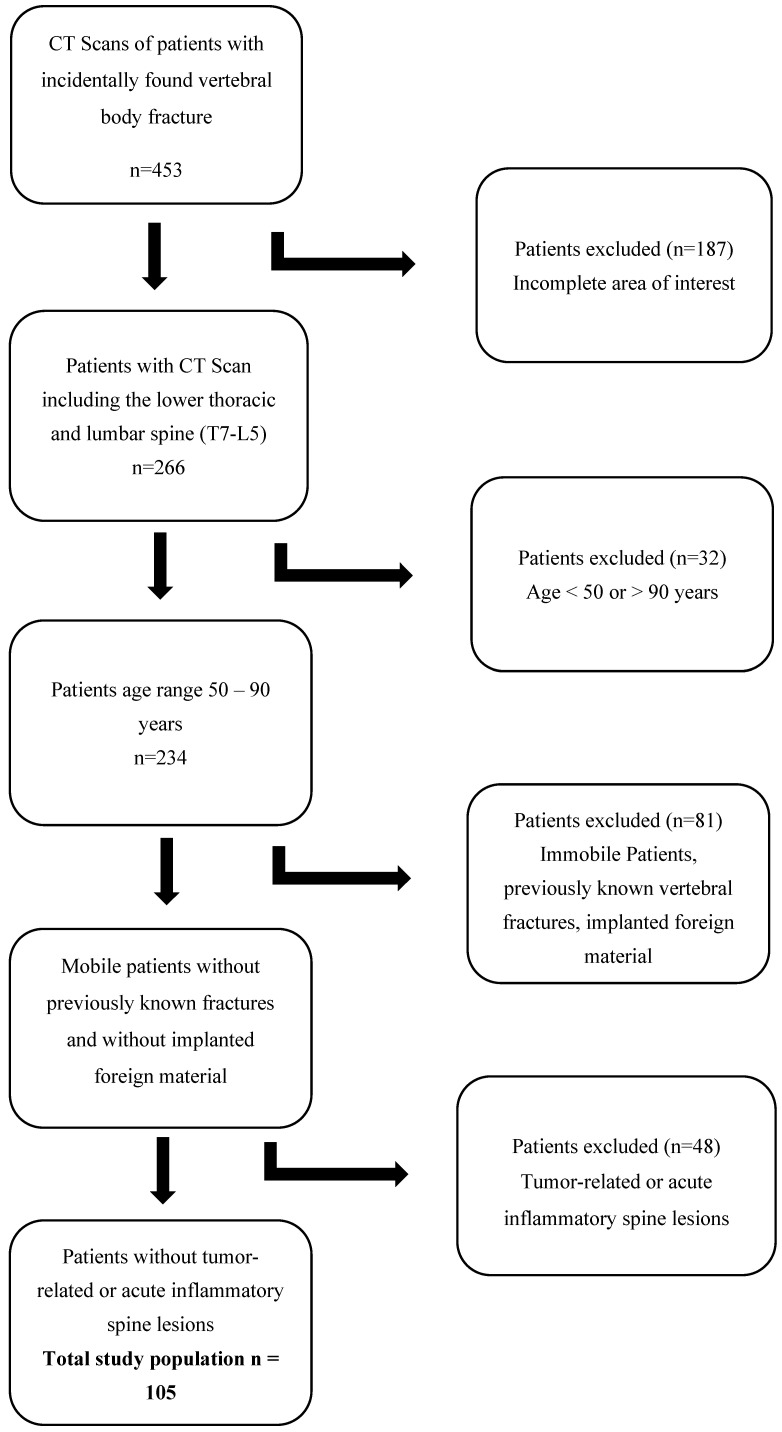
Flow chart of study selection based on the inclusion and exclusion criteria.

Figure 1 shows a flow chart of study selection based on the inclusion and exclusion criteria.

The patients were scanned using multidetector and spectral CT scanners manufactured by Philips Healthcare (IQon Spectral CT) and Siemens Healthineers (Somatom Definition Flash). The two CT scanners were cross-calibrated in several previous studies, where the same combination of CT scanners was already used. Therefore, we decided not to cross-calibrate again, as both CT scanners are also largely comparable. The scans contained volumetric data with a minimum of 2 mm thick slices and multiplanar reconstructions.

### 2.2. Image Analysis

**Figure 2:**
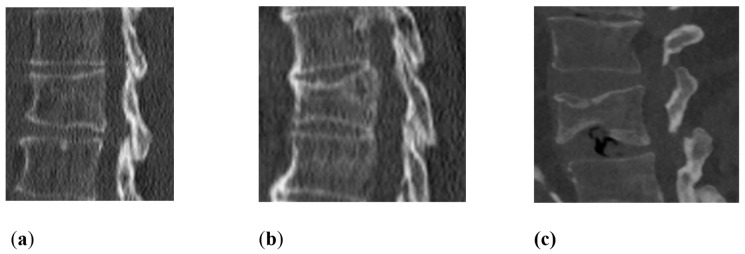
Genant score in sagittal CT scans: (**a**) Grade 1 20–25% height loss. (**b**) Grade 2 26–40% height loss. (**c**) Grade 3 > 40 %.

**Figure 3:**
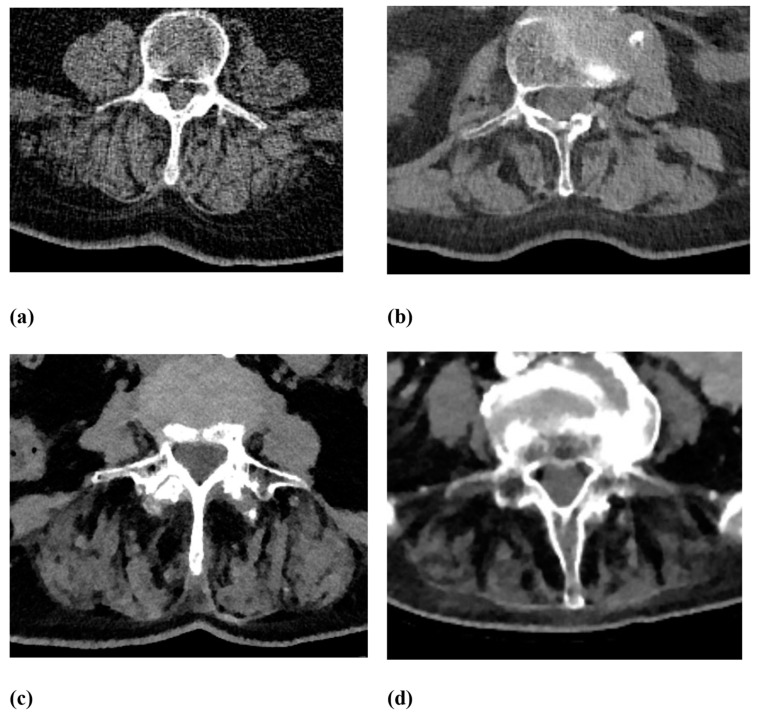
Goutallier score modified for CT scans along the lumbar spine. (**a**) Grade 1 for a few fatty streaks within the muscle. (**b**) Grade 2 for less fat than muscle within the muscle. (**c**) Grade 3 for the same amount of fat and muscle within the muscle. (**d**) Grade 4 for slightly fatter than muscle within the muscle.

**Figure 4:**
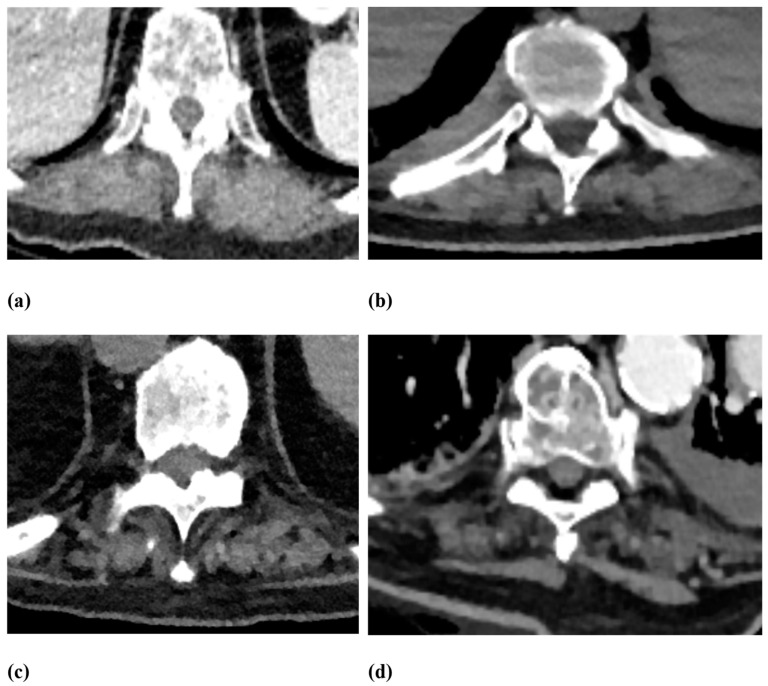
Goutallier score modified for CT scans along the thoracic spine. (**a**) Grade 0 for normal muscle without fat. (**b**) Grade 1 for a few fatty streaks within the muscle. (**c**) Grade 2 for less fat than muscle within the muscle. (**d**) Grade 3 for approximately the same amount of fat and muscle within the muscle.

Osseous and extra-osseous degenerative changes were analyzed in correlation with non-traumatic fractures. Vertebral body fractures were evaluated by separately analyzing two spine levels: the lower thoracic spine (T7–T12) and the lumbar spine (L1–L5). First, non-traumatic vertebral body fractures were identified in the mentioned levels. Then, the vertebral body fractures were classified using the Genant score: Grade 0—no deformity, Grade 1—20–25% height loss, Grade 2—26–40% height loss, and Grade 3—>40% height loss (Figure 2). The classification included wedge, biconcave, and compression deformations [18,19]. Osseous changes contained osteochondrosis, facet joint arthrosis and asymmetry, spondylolisthesis, and spondylolysis. Facet joint arthrosis was classified as normal, mild, moderate, and severe, considering articular surface irregularities, osteophyte extensions, and subchondral sclerosis. Facet joint asymmetry, when comparing the left and right facet joints of each segment, was divided as normal 0–10°, mild 11–30°, moderate 31–60° and severe >60°. Intervertebral osteochondrosis was classified as mild, moderate, and severe using moderated Modic type changes on CT scans, considering disc height, osteophytes, subchondral sclerosis, and vacuum phenomenon. Spondylolisthesis was analyzed using the Meyerding classification, 0% to 25% as Grade I, 25% to 50% as Grade II, 50% to 75% as Grade III, 75% to 100% as Grade IV and >100% as Grade V [20]. Scoliosis was classified as normal 0–10°, mild as 11–25°, moderate as 26–45° and severe as >45°. In addition, Schmorl nodes as common spinal disc herniations were evaluated. Extra-osseous changes contained fatty degeneration and asymmetry of the autochthonous back muscles. These were graded using the Goutallier score 0–4: grade 0 for “normal muscle without fat”, grade 1 for “few fatty streaks within the muscle”, grade 2 for “less fat than muscle within the muscle”, grade 3 for “the same amount of fat and muscle within the muscle”, and grade 4 for “more fat than muscle within the muscle” [21,22] (Figure 3 and Figure 4). In multislice CT scans, the fatty infiltration presented as an apparent decrease in density, often streaking through the entire muscle. A reading protocol for the evaluation was established, and the collected CT scans were analyzed by two experienced radiologists (readers) with findings reached by consensus. The readers were allowed to discuss their findings and had to present one consensus result for each rating. A summary of the classifications used in this study for certain variables has been added to Table 1.

### 2.3. Statistics

Basic variables are given as proportions (%). Descriptive statistics were used to calculate basic variables and proportions. The group values were compared using the Mann–Whitney U-test as indicated. Relative risks (RRs) were calculated using contingency tables. The correlation between the different grades of degenerative changes and the fracture severities was evaluated using Pearson’s correlations (Rs). Thereby, the degeneration grades and fracture severities were transferred into numerical grades (1, 2, 3, 4, and in some cases, 5). The number 1 indicates the lowest and 4/5 the highest grade of degeneration or fracture severity. Statistical significance for all tests was set at *p* < 0.05. Statistical analysis was performed using the IBM-SPSS version 26.0 software package (IBM, Armonk, NY, USA).

## 3. Results

Low-grade fatty infiltration of the autochthonous muscles, including Goutallier 1, was found in 80.9% of the patients, while higher-grade fatty infiltration (excluding Goutallier 1) was found in 24.7% of the patients. Asymmetry of the autochthonous muscles in the lower thoracic and lumbar spine was noted in 37.1% of the patients. Further basic variables of the study are summarized in Table 2. Interesting observations were made when analyzing the results of extra-osseous degenerative changes. There was a high incidence of facet joint anisotropy in the lumbar spine (65.7%) and a relatively low incidence in the thoracic spine (13.3%). Another expected finding was the higher incidence of spondylarthrosis in the lumbar spine (80%) compared to the thoracic spine (31.4%). Schmorl nodes were more frequently noted in the thoracic spine (52.3%) than in the lumbar spine (35.2%).

Fatty deposits in autochthonous muscles are associated with vertebral body fractures in the lower thoracic spine (*p* = 0.005). The relative risk ratio (RR) for the occurrence of these fractures was 4.92 (Table 3). Fatty degeneration of autochthonous muscle also correlated with the severity of vertebral body fractures (Table 4). In contrast, fatty infiltration of the autochthonous muscles was not a significant risk factor for lumbar spine fractures (RR = 2.04; *p* = 0.157). Interestingly, the asymmetry of autochthonous muscles also showed no significant association with vertebral body fractures. All other investigated degenerative changes did not present significant differences between patients with fractured and non-fractured vertebral bodies (Table 3).

Table 3 provides an overview of relative risk ratios (RR) and the significance of patients with fractured vertebral bodies.

Figure 2 shows different grades of vertebral height loss, while Figure 3 and Figure 4 present different grades of fatty degeneration of the autochthonous muscles of the thoracal and lumbar spine.

## 4. Discussion

Degenerative changes in the spine summarize a variety of lesions that can be challenging to address and are often overlooked in radiological reports. Fatty degeneration of the autochthonous back muscles, as an extraosseous change, is proving to have an even more significant impact on the spine than previously thought by significantly correlating to non-traumatic vertebral fractures in this study. Interestingly, the other osseous and extra-osseous degenerative changes did not significantly affect non-traumatic vertebral fractures. These results are unsurprising, as it has been challenging to find significant correlations between osseous changes in the spine to assess specific risk factors. It is well known that degenerative changes in the spine, especially disc degeneration and facet joint arthrosis, and Modic-type changes in the vertebrae correlate with back pain [23,24,25]. Degenerative disc changes have also been reported to correlate with vertebral body fractures, especially in the anterior wall [26,27]. On the contrary, associations between the degenerative changes recorded in this study and vertebral fractures have generally not been found.

The importance of fatty depositions in autochthonous back muscles has been reported in back pain, postural deformity, and intervertebral disc degeneration, leading to patient disability and reduced quality of life [28,29,30]. This study also associated fatty degeneration in autochthonous muscles with non-traumatic vertebral fractures. These fractures can potentially reduce the patient’s mobility and functionality in daily life and, in some cases, may also indicate further surgical treatment [31,32]. In particular, osteoporotic/non-traumatic fractures require a multimodal approach. The most invasive procedure is surgery, where dorsal spondylodesis is well established and constantly improved to achieve correct implantation and the best medical outcome, especially with a perfect pedicle screw position [33]. Besides surgery, medical treatment is well-established to prevent fractures, but several conservative options include strengthening the autochthonous back muscles [34]. Physiotherapy in so-called back schools to increase muscle strength and stability can also help patients with chronic back pain and vertebral fractures [35,36]. Considering that only incidental vertebral fractures were included in this study, the current state of research shows a general trend towards consequential treatment, especially conventional options, as surgery is more often used only after repeated vertebral fractures, ideally in the subacute state or when conventional treatment has shown no effect [37,38,39]. In particular, the evaluation of treatment for underlying osteoporosis shows that it is used more often after an incidental finding of vertebral body fractures, but overall, it is still low [37]. Although our study emphasizes the importance of back exercises for maintaining vertebral body alignment, conventional training and exercises cannot solely prevent non-traumatic vertebral fractures. However, exercises could be counted as one favorable factor for maintaining vertebral body alignment.

Daily radiological reports of CT scans could include grading the quality of the autochthonous back muscles. As shown in Figure 3 and Figure 4, CT scans provided a detailed differentiation of fat deposition in autochthonous back muscles regarding differences in Hounsfield units visualizing streaky fat infiltration. As MRI scans play a leading role in demonstrating muscle quality, it was interesting that CT images could be an additional method of analyzing muscle structure. Conveniently, many CT scans for various indications, especially in larger clinics, will include the spine with autochthonous muscles on the side. Future studies could add quantitative measurements using novel CT imaging techniques, such as spectral CT, to improve and expand image analysis [40]. In the variety of osseous and extraosseous degenerative changes in the spine, these results may improve selection for radiologists, especially since in our study, all other degenerative changes showed no significance for non-traumatic fractures.

Given the Genant score, most of the vertebral fractures in this study had a wedge deformity. As these can have various treatment options, such as the consideration of conventional therapy with back braces or surgical therapy with balloon kyphoplasty, their detection has implications for the patient. The correlation demonstrated in this study may help sensitize radiological reports to fractures in patients with fatty degenerated back muscles.

This study has some limitations. MRI is usually the primary imaging technique used to assess muscle quality. Although CT scans were adequate for this study, MRI can determine muscle structure to the degree that CT scans cannot. Accordingly, the Goutallier score for grading fatty deposits in muscle was developed for MRI and used in this study in a modified form. In addition, the Goutallier score was initially developed for rotator cuff degenerations, although it is commonly used for other muscle groups, particularly autochthonous back muscles [41]. Considering that our main exclusion criterion was incomplete visualization of the area of interest, it must be noted that this circumstance reduces the general applicability of this study. In addition, the age range and the exclusion of any tumor or inflammatory spinal lesions further limit the generalizability. Additionally, we cannot entirely verify whether the fatty muscle degeneration or the fracture was first. We performed a cross-sectional study, which did not include any second time point. Further studies could clarify this question by performing larger cohort or case–control studies. A further limitation is the lack of clinical information on patients, particularly in relation to back pain, and the lack of information on reasons for referral. Imaging alone could only partially assess patient status and not highlight individual clinical circumstances. However, as the study’s main aim was to compare the relevance of degenerative changes in the spine with clearly visible vertebral fractures, the clinical data only partially influence this collective. Furthermore, our patient numbers could be much higher. Future studies should investigate our findings in larger cohorts.

## 5. Conclusions

Fatty degeneration of the autochthonous spinal musculature is significantly associated with non-traumatic fractures of the lower thoracic spine. Consecutively, radiologists should be aware of potential spine fractures when identifying patients with the mentioned degenerative changes. Our findings suggest that the clinical focus should be shifted further towards strengthening the autochthonous back muscles.

## Figures and Tables

**Table 1:** Classification of each variable.

Vertebral body fractures	Genant Score: Grade 0: no deformity; Grade 1: 20–25% height loss; Grade 2: 26–40% height loss; Grade 3: >40% height loss (for more detail, see Figure 2).
Fatty degeneration of the autochthonous back muscles	Goutallier score 0–4: Grade 0 for “normal muscle without fat”; Grade 1 for “few fatty streaks within the muscle”; Grade 2 for “less fat than muscle within the muscle”; Grade 3 for “the same amount of fat and muscle within the muscle”; Grade 4 for “more fat than muscle within the muscle” (for more details, see Figure 3 and Figure 4).
Asymmetry of the autochthonous back muscles	Normal: symmetrical visual muscle volume; Mild: <10% visual muscle volume difference; Moderate: <30% visual muscle volume difference; Severe: >50% visual muscle volume difference.
Facet joint arthrosis	Normal: sharp articular surface, no osteophytes, and no subchondral sclerosis. Mild: slightly irregular articular surface, small osteophytes, and no subchondral sclerosis; Moderate: irregular articular surface, osteophytes, and incipient subchondral sclerosis; Severe: irregular articular surface, large osteophytes, advanced subchondral sclerosis, and vacuum phenomenon.
Facet joint asymmetry	Normal: 0–10°; Mild: 11–30°; Moderate: 31–60°; Severe: >60°.
Intervertebral osteochondrosis	Normal: preserved disc height, no osteophytes, and no subchondral sclerosis; Mild: preserved disc height, small osteophytes, and no subchondral sclerosis; Moderate: reduced disc height, ventral and dorsal osteophytes, and mild subchondral sclerosis; Severe: collapsed disc, vacuum phenomenon, osteophytes, syndesmophytes, and advanced subchondral sclerosis.
Spondylolisthesis	Meyerding classification: Grade I: 0% to 25%; Grade II: 25% to 50%; Grade III: 50% to 75%; Grade IV: 75% to 100%; Grade V: >100%.
Scoliosis	Normal: 0–10°; Mild: 11–25°; Moderate: 26–45°; Severe: >45°.

**Table 2:** Basic variables of the study population.

Criteria	Study Patients (n = 105; Mean Age = 68.7 Years)
	%
Vertebral body fractures	23.8
At least one thoracic vertebral body fracture	16.2
At least one lumbar vertebral body fracture	19
Fatty depositions of the autochthonous muscles at least Goutallier 1	80.9
Fatty depositions of the autochthonous muscles at least Goutallier 2	24.7
Asymmetry of the autochthonous muscles	37.1
At least moderate osteochondrosis of the lower thoracic spine	64.7
At least moderate osteochondrosis of the lumbar spine	68.5
At least moderate spondylarthrosis of the lower thoracic spine	31.4
At least moderate spondylarthrosis of the lumbar spine	80
At least mild facet joint anisotropy of the lower thoracic spine	13.3
At least mild facet joint anisotropy of the lumbar spine	65.7
At least moderate facet joint anisotropy of the lumbar spine	18
Spondylolisthesis of the lumbar spine	9.5
Spondylolysis of the lumbar spine	4.7
Short vertebral height deformity	2.8
Schmorl nodes of the lower thoracic spine	52.3
Schmorl nodes of the lumbar spine	35.2
Scoliosis	20.9

**Table 3:** Statistical analysis of potential risk factors for non-traumatic thoracal and lumbar spine fractures. The data are divided into the lower thoracic and lumbar vertebral body fractures. Each of these two sections shows relative risks (RRs) and *p*-values (fractured versus non-fractured).

	Thoracic Vertebral Body Fracture		Lumbar Vertebral Body Fracture	
Risk Factor	Relative Risk (RR)	*p*-Value	Relative Risk (RR)	*p*-Value
Osteochondrosis thoracic spine	0.94	0.314	0.94	0.327
Osteochondrosis lumbar spine	0.96	0.375	0.69	0.76
Spondylarthrosis thoracic spine	0.83	0.067	0.88	0.343
Spondylarthrosis lumbar spine	1.04	0.431	0.46	0.527
Fatty degeneration autochthonous muscle thoracic and lumbar spine	4.92	0.005	2.04	0.157
Asymmetry autochthonous muscle thoracic and lumbar spine	1.54	0.439	0.732	0.444
Spondylolysis lumbar spine	0.93	0.268	0.93	0.271
Spondylolisthesis lumbar spine	0.55	0.576	0.88	0.109
Facet joint anisotropy thoracic spine	2.44	0.197	2.82	0.09
Facet joint anisotropy lumbar spine	0.95	0.18	0.96	0.589
Short vertebral height thoracic spine	1.92	0.232	0.82	0.310
Short vertebral height, lumbar spine	1.35	0.576	0.73	0.394
Schmorl nodes thoracic spine	1.92	0.232	1.35	0.576
Schmorl nodes lumbar spine	1.54	0.394	1.67	0.31
Scoliosis	0.93	0.907	1.19	0.775

**Table 4:** Statistical analysis of the correlations of degeneration grades (as indicated in the methods) to the severity of non-traumatic fractures. Pearson’s correlations (R) and the according *p*-values are shown for each section.

	Thoracic Vertebral Body Fracture		Lumbar Vertebral Body Fracture	
Risk Factor	Correlation (R)	*p*-Value	Correlation (R)	*p*-Value
Osteochondrosis thoracic spine	0.02	0.848	−0.08	0.43
Osteochondrosis lumbar spine	−0.04	0.718	0.05	0.625
Spondylarthrosis thoracic spine	0.16	0.104	0.11	0.257
Spondylarthrosis lumbar spine	0.12	0.223	0.03	0.767
Fatty degeneration autochthonous muscle thoracic and lumbar spine	0.34	<0.001	0.18	0.066
Asymmetry autochthonous muscle thoracic and lumbar spine	0.15	0.130	0.16	0.124
Facet joint anisotropy thoracic spine	0.14	0.162	-0.08	0.397
Facet joint anisotropy lumbar spine	0.16	0.107	-0.07	0.471
Scoliosis	0.068	0.49	-0.015	0.883

## Data Availability

Research data cannot be shared due to ethical issues.
